# Phenotype and multipotency of rabbit (*Oryctolagus cuniculus*) amniotic stem cells

**DOI:** 10.1186/s13287-016-0468-z

**Published:** 2017-02-07

**Authors:** Jéssica Borghesi, Lara Carolina Mario, Ana Claudia Oliveira Carreira, Maria Angélica Miglino, Phelipe Oliveira Favaron

**Affiliations:** 1 Department of Surgery, School of Veterinary Medicine and Animal Science, University of Sao Paulo, Sao Paulo, SP Brazil; 2 NUCEL (Cell and Molecular Therapy Center) and NETCEM (Center for Studies in Cell and Molecular Therapy), School of Medicine—Chemistry Institute, Biochemistry Department, Sao Paulo University, Sao Paulo, SP Brazil; 3 Orlando Marques de Paiva, 87, Cidade Universitária, Sao Paulo, SP 05508-270 Brazil

**Keywords:** Mesenchymal stem cells, Amniotic membrane, Rabbit, Proliferative capacity

## Abstract

**Background:**

Stem cells are capable of unlimited self-renewal and are able to remain undifferentiated for extended periods of time prior to their differentiation into specific cell lineages. Because of the issues (ethical and religious) involved in the use of embryonic stem cells and the limited plasticity of adult stem cells, an alternative cell source could be foetal stem cells derived from extra-embryonic tissue, which are highly proliferative, grow in vitro and possess interesting immunogenic characteristics. As a result, the amniotic membrane of several species has been studied as an important new source of stem cells.

**Methods:**

Here, we cultured and characterized mesenchymal progenitor cells derived from the rabbit amniotic membrane, and investigated their differentiation potential. In total, amniotic membranes were collected from eight rabbit foetuses and were isolated by the explant technique. The obtained cells were cultured in DMEM-HIGH glucose and incubated at 37 °C in a humidified atmosphere with 5% CO_2_.

**Results:**

The cells adhered to the culture plates and showed a high proliferative capacity with fibroblast-like morphologies. The cells showed a positive response for markers for the cytoskeleton, mesenchymal stem cells and proliferation, pluripotency and haematopoietic precursor stem cells. However, the cells were negative for CD45, a marker of haematopoietic cells. Furthermore, the cells had the capacity to be induced to differentiate into osteogenic, adipogenic and chondrogenic lineages. In addition, when the cells were injected into nude mice, we did not observe the formation of tumours.

**Conclusions:**

In summary, our results demonstrate that multipotent mesenchymal stem cells can be obtained from the rabbit amniotic membrane for possible use in future cell therapy applications.

## Background

In recent years, there has been growing interest in obtaining stem cells derived from extra-embryonic tissues because they possess considerable expansion and proliferative capacities in vitro, which favours the establishment of cell line banks. In addition, those human tissues are routinely discarded after birth, and their use does not involve significant ethical or religious problems [1–3].

Stem cells derived from foetal membranes and placental tissues maintain the characteristics of embryonic tissues, including the expression of markers associated with embryonic stem cells, such as Oct-4 and Nanog, and a high proliferative capacity in vitro [4–6] without the risk of tumourigenesis [7, 8]. This is in contrast to embryonic cells, where histocompatibility problems increase the potential for tumourigenicity [1] and there are ethical considerations involved in their use.

The amniotic membrane in particular has been the focus for researchers [9, 10]. The membrane arises early from amnioblasts derived from the epiblast, which could favour the potential for high pluripotency, as has been confirmed by the expression of markers such as Sox-2, Nanog, Oct-4 and Rex-1 [11, 12]. However, the potential applications of amniotic membrane cells are not due only to their pluripotency but also to their immunogenic characteristics [13, 14]. Various transplant studies and grafts performed using human amniotic membrane-derived cells have shown a lack of an immune response [15–17], which could be explained by the immunomodulating properties of foetal membranes that are involved in maintenance of maternal–foetal tolerance [10]. Another important factor is that these cells show major histocompatibility complex (MHC) class I but not MHC class II expression [16, 18, 19]. Because of these characteristics, the amniotic membrane has emerged as an important new source of stem cells in different species including human [20–22], horse [11, 23, 24], sheep [25], cat [7, 26], dog [3, 5] and mouse [27] (Table 1).

**Table 1:** Cells derived from amniotic membranes

Species	Cell types	Markers expressed	Differentiated lineages	References
Human	Epithelial and mesenchymal	CD90, CD44, CD73, CD116, CD117, CD105, CD29, SSEA-4, STRO-1	Osteogenic, chondrogenic, adipogenic, myogenic, neurogenic	[10, 20–22, 29, 73]
Cat	Epithelial	SSEA-4, CD34 Oct-4, Nanog, CD44, CD116	Osteogenic, chondrogenic, adipogenic, neurogenic	[7, 26]
Dog	Mesenchymal	CD90, CD105	Osteogenic, chondrogenic, adipogenic	[3, 5]
Horse	Mesenchymal	CD19, CD20, CD28, CD31, CD38,CD41, CD44, CD62, CD90, CD105, CD200	Osteogenic, chondrogenic, adipogenic	[11, 23, 24, 49]
Sheep	Epithelial	Oct-4, SSEA-1, SSEA-3, SSEA-4, TRA-1-60 and TRA-1-81, Nanog	Neurogenic, osteogenic	[25, 48, 74]
Mouse	Mesenchymal	CD90, CD29, Vimentin	Osteogenic, adipogenic, neurogenic, hepatogenic	[27]

Characteristics related to the morphologies, phenotypes and differentiation potential of stem cells derived from amniotic membranes of different species

Parolini et al. [10], in their studies of the human amniotic membrane, reported two cell types: epithelial cells derived from the ectoderm; and mesenchymal cells derived from the mesoderm. The epithelial cells derived from the amniotic membrane were isolated by the manual separation of the amnion from the chorion followed by trypsin digestion. In culture, these cells express epithelial markers such as cytokeratin [12], the embryonic cell markers SSEA-4 and TRA-1-60, and the pluripotency markers Oct-4, Sox-2 and Nanog, but do not express mesenchymal markers [19, 28]. Moreover, mesenchymal cells were isolated from the amniotic membrane by the mechanical separation of the amnion and the chorion followed by digestion with collagenase [10]. In culture, these cells express mesenchymal markers such as CD73, CD90 and CD105, but do not express the haematopoietic cell markers CD34 and CD45 [7, 17, 29, 30].

In humans, limitations regarding the collection of amniotic membranes (e.g. their availability only at delivery, except in some abortion cases) could restrict our ability to study the true potential of this tissue to be a source of stem cells. In this regard, the use of animal models is essential to further our understanding of the plasticity of this membrane. In addition, the characterization of new species as experimental models is of fundamental importance because experimental research and preclinical testing are prerequisites for further clinical use and as such are extremely relevant in human medicine [31]. In this context, rabbits have served as important experimental models due to their advantages related to behaviour, low maintenance costs and short period of pregnancy (32 days) [32, 33]. Moreover, rabbits are more phylogenetically similar to primates than are rodents and possess a more diverse genetic background, making them more suitable for use as experimental models [34].

Considering the advances in veterinary medicine in the last years, as well as the perspectives of an applied translational medicine, stem cells have an important role in this context. Because of the problematic aspects related to obtaining samples of human placental tissues from early to middle gestation, animal models may solve important gaps in our knowledge, especially rabbits which share several morphological and physiological characteristics with humans, in addition to the apparent success in obtaining, culturing and utilizing different lineages of progenitor cells from rabbit tissues [35–37]. In this way, this study aimed to isolate, characterize and differentiate progenitor cells obtained from the rabbit amniotic membrane at 16 days of gestation.

## Methods

### Animals

In total, eight rabbit foetuses were used at 16 days of gestation. The animals were derived from the slaughterhouse of the Faculty of Animal Science and Food Engineering, University of São Paulo, FZEA-USP, which is located in Pirassununga, SP, Brazil. The project was approved by the bioethics commission of the School of Veterinary Medicine and Animal Science-USP (Protocol number 1891200514).

### Isolation, culture and morphological analysis of cells derived from the amniotic membrane

After their collection, fragments of the amniotic membrane were deposited in Petri dishes (catalogue number 3296; Corning, NY, USA) and were washed five times with PBS and penicillin–streptomycin 5% in a laminar flow. Explants from the amniotic membranes were placed in 100-mm diameter culture plates (catalogue number 3296; Corning) with 7 ml of culture medium. Three culture media were tested—DMEM-high glucose (DMEM-HIGH, catalogue number BR30003-05; LGC Biotecnologia, Cotia, SP, Brazil), DMEM-F12 (1:1) (catalogue number BR30004-05; LGC Biotecnologia) and MEM/ALPHA (catalogue number BR3007-05; LGC Biotecnologia)—and each condition was supplemented with 15% foetal bovine serum (FBS, catalogue number BR330110-01; LGC Biotecnologia), 1% streptomycin/penicillin, 1% essential amino acids (catalogue number BR30238-01; LGC Biotecnologia) and 0.05% sodium pyruvate (catalogue number S8636; Sigma, St. Louis, MO, USA). Plates were maintained in an incubator at a temperature of 37 °C with a humidified atmosphere containing 5% CO_2_. After culturing for 24 hours, the first few adherent cells were observed, and the cell adhesion medium was changed every 48–72 hours. When cells reached 80% confluence, they were subjected to trypsinization for expansion. For this, cells were washed with PBS and then 0.25% trypsin (catalogue number BR30042-01; LGC Biotecnologia) was added. After 5 minutes at 37 °C, the trypsinization was stopped by adding the same volume of culture medium. The cells were then centrifuged for 5 minutes at 1200 rpm. Morphological analysis of the cells was performed every 3 days using an inverted microscope (Nikon Eclipse TS-100), and the experiments were performed in passages 4 and 8.

### Cryopreservation test

Aliquots of 1 × 10^6^ cells derived from the amniotic membrane were frozen for the following experiments. The freezing medium was comprised of 90% FBS and 10% dimethyl sulfoxide (DMSO, catalogue number 13-0091.01; LGC Biotecnologia). The cells were subcultured and resuspended in 1.5 ml freezing medium and then equally distributed into cryotubes (catalogue number 430488; Corning). The cryotubes were transferred to a freezer at −80 °C overnight, and after 24 hours the cryotubes were transferred to liquid nitrogen. For experiments, the cells were subjected to rapid thawing in a water bath at 37 °C.

### Scanning electron microscopy

Cells were cultivated on glass coverslips, and after culture the cells were fixed in 2.5% glutaraldehyde, washed in 0.1 M pH 7.4 phosphate buffer and post-fixed in 1% osmium tetroxide followed by continuous dehydration in ethanol (70%, 80%, 90%, 95% and 100%). Finally, the cells were dehydrated to the dry critical point (Balzers CPD 020). Subsequently, the samples were placed in a metallic support for gold lining (“sputtering” Emitech K550), and the analysis was performed in a scanning electron microscope (ME Leo 435 VP).

### Colourimetric assay (MTT)

The colourimetric assay (MTT) [38] consists of the reduction of the yellow-coloured 3-((4,5-dimethylthiazol-2-yl)-2,5-diphenyltetrazolium bromide) (MTT) compound by the mitochondrial dehydrogenase enzyme (component complex II of the Krebs cycle), which is present only in viable cells. The experiment was conducted every 48 hours for 12 days (288 hours) to analyse the proliferation of rabbit amniotic membrane cells. On each day of the experiment, approximately 1 × 10^3^ cells were plated in 96-well plates in a volume of 210 μl/well. Next, the culture medium was removed, and 100 μl of new medium with 10 μl of MTT solution A (catalogue number V-13154; Life Technologies, Carlsbad, CA, USA) was added. The solution was incubated for 4 hours at 37 °C, and after this period 100 μl of Solution B (catalogue number V-13154; Life Technologies, Carlsbad, CA, USA) was added, and the cultures were maintained for an additional 4 hours at 37 °C. The analysis was then performed using a spectrophotometer (MQuant–Bio Tek Instruments, VT, USA).

### Flow cytometry

Immunophenotypic analysis was performed using flow cytometry. For this, 1 × 10^5^ cells/ml were trypsinized, centrifuged and fixed in 10% paraformaldehyde. The cells were then incubated with primary antibodies (dilution 1:100) for 30 minutes at 4 °C. After this period, the cells were incubated with the secondary antibody (dilution 1:500) for 30 minutes at 4 °C. Finally, the cells were washed and analysed by a BD FACSariaIIu flow cytometer (Becton Dickinson, San Jose, CA, USA). Controls were performed using unmarked cells exposed only to the non-specific secondary antibody. For each sample, 10,000 events were counted. The primary antibodies used were as follows: Nanog (n-17, sc30331), Sox-2 (sc-17320), vimentin (sc-73259), cytokeratin 18 (RGE53, sc-32329), Stro-1 (sc-47733), PCNA-3 (sc-46), β-tubulin (sc-47751), SSEA-4 (sc-59368), TRA-1-60 (sc-21705) and CD73 (sc-14684) (all purchased from Santa Cruz Biotechnology, Inc., Europe); in addition to CD105 (ab53321; Abcam, Cambridge, UK), CD117 (A4502; Dako Cytomation, Carpinteria, CA, USA) and CD45 (MHCD4501; Invitrogen, Carlsbad, CA, USA). For nuclear antibodies such as PCNA-3, Nanog and Sox-2 we added 0.1% of triton (catalogue number 13-1315-05; LGC Biotecnologia) in order to permeabilize the cell membranes.

### Total RNA isolation, cDNA synthesis and real-time reverse transcription PCR

Total RNA was extracted from amniotic cells using the Trizol manufacturer’s (catalogue number: 12183555 Invitrogen, Carlsbad, CA, USA) protocol, treated with DNAseI (Thermo Scientific), and 1 μg was reverse-transcribed to first-strand cDNA and subjected to quantitative real-time reverse transcription PCR (RT-qPCR) according to Fratini et al. [39]. The primer sequences (*Lin28a*, *c-myc*, *GAPDH*) are presented in Table 2. *GAPDH* was used as the reference gene. Relative expression levels of *Lin28a* and *c-myc* were calculated according to the Pfaffl model [40].

**Table 2:** List of primer sequences used for RT-qPCR analysis in this study

Gene name	Gene	Direction per gene	Accession number
Lin-28 homolog A	*Lin28a*	Forward 5´-GCACCAGAGTAAGCTGCACA	XM_002716102.3
		Reverse 5´-GGCGGACTTCTTGAAGGTGA	
c-Myc	*v-myc* avian myelocytomatosis viral oncogene homolog	Forward 5´-TCTGCTCTCCTCCAACGAGT	NM_001319575.1
		Reverse 5´-TTGTTCTTCCTCAGAGTCGCT	
Glyceraldehyde-3-phosphate dehydrogenase	*GAPDH*	Forward 5´-AAGGGTCATCATCTCAGCCC	NM_001082253.1
		Reverse 5´-TGCGTTGCTGACAATCTTGAG	

*RT-qPCR* real-time reverse transcription PCR

Statistical analysis was performed using GraphPad Prisma software (version 6.01) with one-way ANOVA followed by Tukey’s test for post-hoc comparison. *p* < 0.05 was considered statistically significant. The results were presented as mean ± SEM.

### Differentiation assays

For osteogenic and adipogenic differentiation, 1 × 10^4^ cells were plated in 24-well plates. For osteogenic induction 48 hours later, the medium was replaced with osteogenic induction medium, which was comprised of DMEM-HIGH supplemented with 7.5% FBS, 100 μM ascorbic acid (Invitrogen), 100 mM β-glycerophosphate (Sigma) and 0.1 mM dexamethasone (Decadron, Schering-Plough, SP, Brazil). On day 21, cells were washed with PBS and fixed with 4% paraformaldehyde. Finally, the cells were stained by Von Kossa and Alizarin Red stains. Adipogenic differentiation was induced by adding DMEM-HIGH supplemented with 7.5% FBS, 10 μg/ml insulin (Sigma-Aldrich, St. Louis, MO, USA), 100 μM indomethacin (Sigma-Aldrich) and 1 mM dexamethasone (Decadron). After approximately 21 days, the cells were washed with PBS, fixed with 4% paraformaldehyde for 15 minutes and stained with Oil Red. For chondrogenic differentiation, 1 × 10^6^ cells were resuspended in inductor medium (StemPro® Chondrogenesis Differentiation Kit, catalogue number A 10071–01; GIBCO Invitrogen, Carlsbad, CA, USA) in a 15-ml conical polypropylene tube and then centrifuged at 1200 rpm for 5 minutes. The obtained cells were maintained at 37 °C in a humidified atmosphere with 5% CO_2_, and the culture medium was changed twice a week. After 21 days, cell aggregates were embedded in paraffin and sectioned at 3–4 mm in an automatic microtome (RM2165; LEICA). The slides were then stained using Picrosirius and Masson’s trichrome.

### Tumourigenicity assay

Nude mice was donated by the bioterium of FMVZ-USP. To determine the tumourigenic potential, 1 × 10^6^ cells were injected intramuscularly into the dorsal region of two nude mice. The mice were maintained under specific pathogen-free conditions using an Isorack and maintained for 8 weeks on sterile food and water ad libitum in the bioterium of the School of Veterinary Medicine and Animal Science, University of Sao Paulo, Brazil. Every week, the animals were evaluated clinically to identify possible tumour development. After 8 weeks, the animals were euthanized using a CO_2_ chamber following the principles of the bioethics committee of FMVZ-USP. Samples from the kidney, lung, liver, heart and spleen were collected and fixed in 4% paraformaldehyde. Tissues for histological examination were embedded in paraffin and sectioned at 5 μm, stained with haematoxylin and eosin (HE) and analysed using an Olympus light microscope (CX 31 RBSFA).

## Results

### Culture and morphology of amniotic membrane cells

**Fig. 1:**
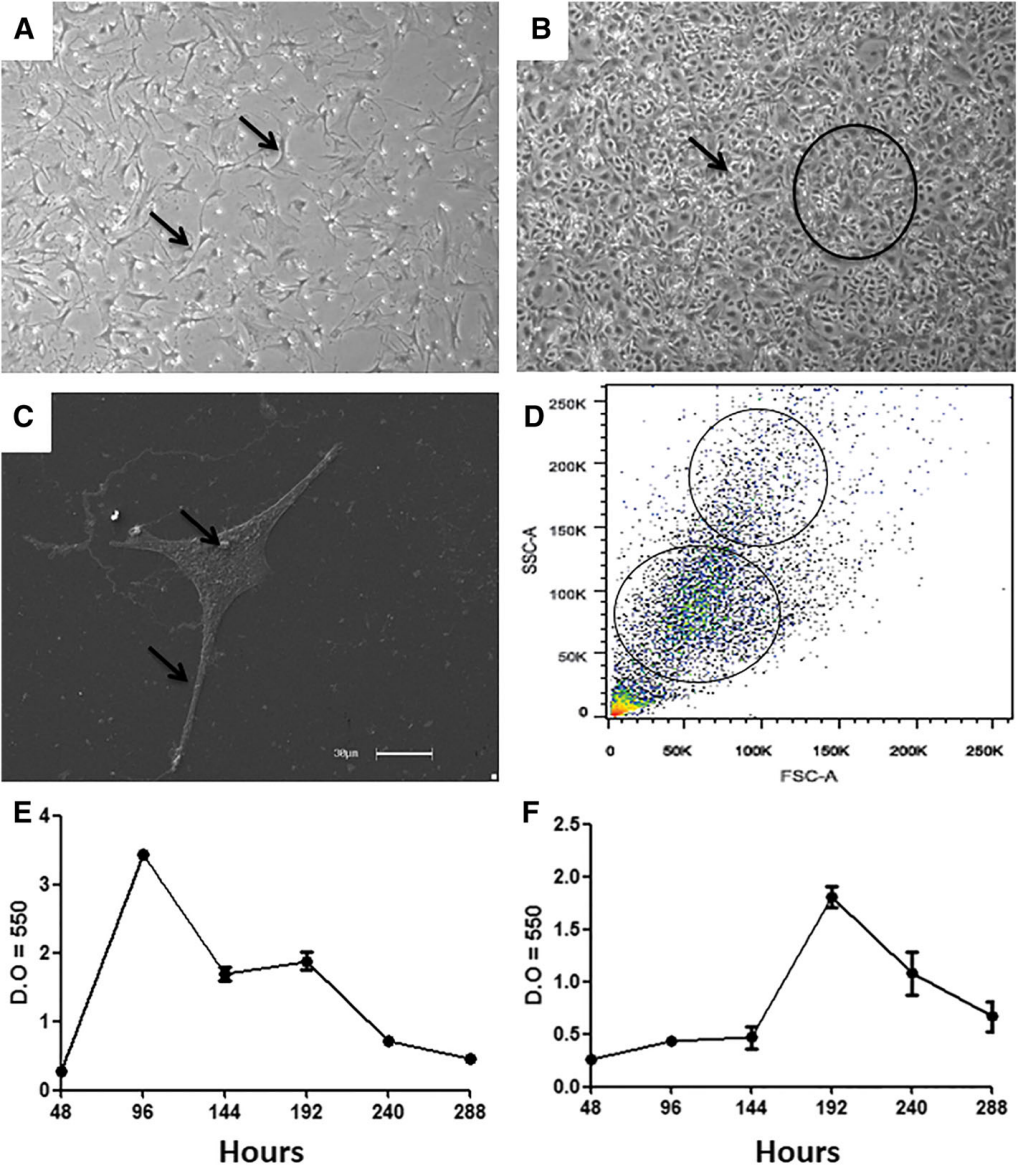
Morphology and viability of rabbit amniotic-derived stem cells. **a** Cells cultured for 24 hours. Fibroblast-like cells adhered to the dish (*arrows*); 20× resolution. **b** Cells cultured for 72 hours. Intense contact between the cells (*arrow*) in high confluence (*circle*); 4× resolution. **c** Scanning electron microscopy. Cells during the fourth passage maintain a fibroblast-like morphology during the passages (*arrows*) with an elongated cytoplasm and central nucleus. **d** Flow cytometry analysis. Two distinct populations (*circles*) with different sizes and granularities. **e**, **f** Cellular metabolism testing during passages 4 and 8, respectively, by the MTT colourimetric method. Increased viability of the passage 4 cells. *FSC* Forward Scatter, *SSC* Side Scatter, *D.O* density optic

For the primary cultures, three different culture media with different glucose concentrations were tested: MEM/ALPHA, DMEM-F12 (1:1) and DMEM-HIGH, containing 5.6 mM, 17.6 mM and 25 mM glucose concentrations, respectively. After testing the three culture media, all of which were supplemented with 15% FBS, the most satisfactory results relating to increased cell adhesion, growth, expansion, morphology and viability of the rabbit amniotic membrane cells were obtained using the DMEM-HIGH medium. After 24 hours of culture, we were able to observe the first adherent cells in the culture plates (Fig. 1a). Every 3 days, cells reached 80% confluence, thereby generating expansion cultures (Fig. 1b). Morphologically, cells had a fibroblast-like shape with an elongated cytoplasm and central spherical nucleus (Fig. 1c). In addition, the cultures remained homogeneous after thawing, with a predominance of cells that maintained satisfactory growth and fibroblast-like morphologies. The cultures were maintained until passage number 8, after which they were analysed by flow cytometry, during which we observed two different cell populations with different sizes and granularities (Fig. 1d).

### Colorimetric assay (MTT)

During the evaluation of the cellular metabolism of passage 4 rabbit amniotic membrane cells grown in DMEM-HIGH, we noted an increase in their metabolic activity during early growth that lasted until the 4th day of the trial and was followed by a decrease that was maintained until the 6th day. The cells then continued to grow until the 8th day, after which the growth rate decreased again until the 12th day (Fig. 1e). In contrast, the metabolic analysis of passage 8 cells demonstrated a low growth rate until the 6th day, which was followed by continued growth until the 8th day and subsequently a steady decrease until the 12th day (Fig. 1f).

### Immunophenotyping

Similar results for nearly all markers were observed for the flow cytometry analysis of rabbit amniotic cells during passages 4 and 8.

**Fig. 2:**
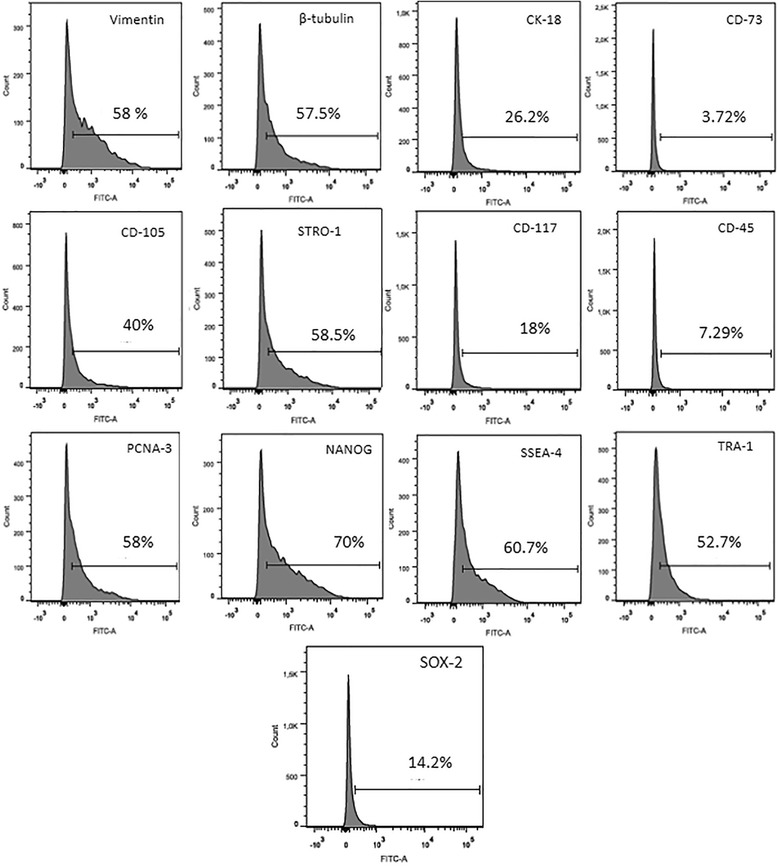
Immunophenotyping of rabbit amniotic cells at passage 4 analysed by flow cytometry. Significant levels of expression for cytoskeletal markers (vimentin, cytokeratin, and β-tubulin) and mesenchymal cell markers (CD105 and Stro-1) and insignificant CD73 expression. Low levels of expression for markers of haematopoietic stem cell precursors and haematopoietic cells (CD117 and CD45, respectively). Significant levels of expression for markers of proliferation (PCNA-3) and pluripotency (Nanog, SSEA-4, Tra-1, and Sox-2). *FITC* fluorescein isothiocyanate

The analysis of rabbit amniotic membrane cells during passage 4 showed high levels of expression for cytoskeletal markers such as vimentin (58%), cytokeratin 18 (57.5%) and β-tubulin (26.2%). Mesenchymal markers such as CD105 (40%) and Stro-1 (58.5%) were also highly expressed, while CD73 (3.72%) was not. There was a low level of expression for the haematopoietic stem cell precursor marker CD117 (18%) and for the haematopoietic cell marker CD45 (7.29%). PCNA-3, a marker of proliferation, was highly expressed (58%), as were the pluripotency markers Nanog (70.1%), SSEA-4 (60.7%) and TRA-1 (52.7%), while there was a lower level of expression for SOX-2 (14.2%) (Fig. 2).

**Fig. 3:**
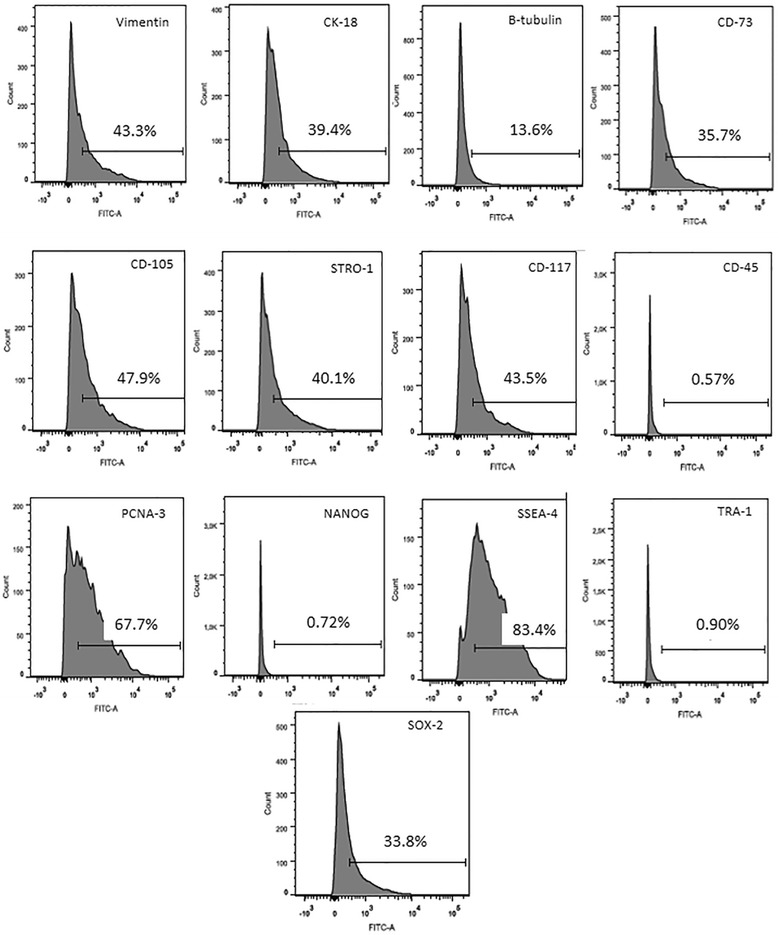
Immunophenotyping of rabbit amniotic cells at passage 8 analysed by flow cytometry. Note the expression levels of cytoskeletal markers (vimentin, cytokeratin and β-tubulin) and mesenchymal cell markers (CD73, CD105 and Stro-1). CD117 (a marker of haematopoietic stem cell precursors) was highly expressed, while CD45 (a marker of haematopoietic cells) was not expressed. There were also significant levels of expression for the proliferation marker PCNA-3 and the pluripotency markers SSEA-4 and Sox-2, while other pluripotency markers (Nanog and TRA-1) were not expressed. *FITC* fluorescein isothiocyanate

At passage 8, the cells demonstrated high levels of the cellular cytoskeleton markers vimentin (43.3%) and cytokeratin 18 (39.4%) but showed low levels of β-tubulin (13.6%) expression. Mesenchymal markers, including CD73 (35.7%), CD105 (47.9%) and Stro-1 (40.1%), were expressed at significant levels. CD117 was also highly expressed during this passage (43.5%), unlike the haematopoietic cell marker CD45 (0.57%) which was insignificantly detected. PCNA-3, a marker of proliferation, was highly expressed (67.7%). Pluripotency markers such as SSEA-4 (83.4%) and Sox-2 (33.8%) were also highly expressed, in contrast to Nanog (0.72%) and Tra-1 (0.90%) which did not show significant levels of expression (Fig. 3).

### RT-qPCR analysis

**Fig. 4:**
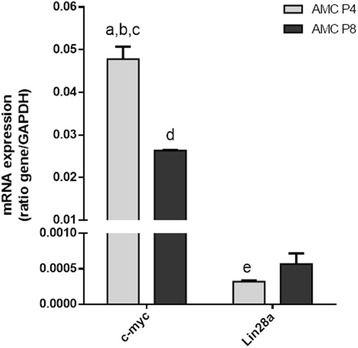
RT-qPCR analysis of amniotic cells (AMC) at passages 4 and 8 (*P4* and *P8*). *X* axis, mRNA expression of the gene as the ratio of target gene/*GAPDH*. ANOVA test followed by Tukey’s test for post-hoc comparison were used for statistical analysis. Results presented as mean ± SEM. Letters show statistically significant expression differences: *a*, *c-myc* P4 vs *Lin28a* P4 (*p* < 0.0001); *b*, *c-myc* P4 vs *c-myc* P8 (*p* < 0.01); *c*, *c-myc* P4 vs *Lin28a* P8 (*p* < 0.0001); *d*, *Lin28a* P4 vs *Lin28a* P8 (*p* < 0.001); *e*, *c-myc* P8 vs *Lin28a* P8 (*p* < 0.001)

To explore the expression levels of pluripotency markers *c-myc* and *Lin28a* in amniotic cells from different passages (passages 4 and 8), RT-qPCR analysis was performed. The results showed that the average level of *c-myc* was increased in amniotic cells in passage 4 in relation to passage 8 (Fig. 4). *Lin28a* had a detectable increase of expression in amniotic cells isolated from passage 8 compared with passage 4 (Fig. 4).

### Differentiation assays

**Fig. 5:**
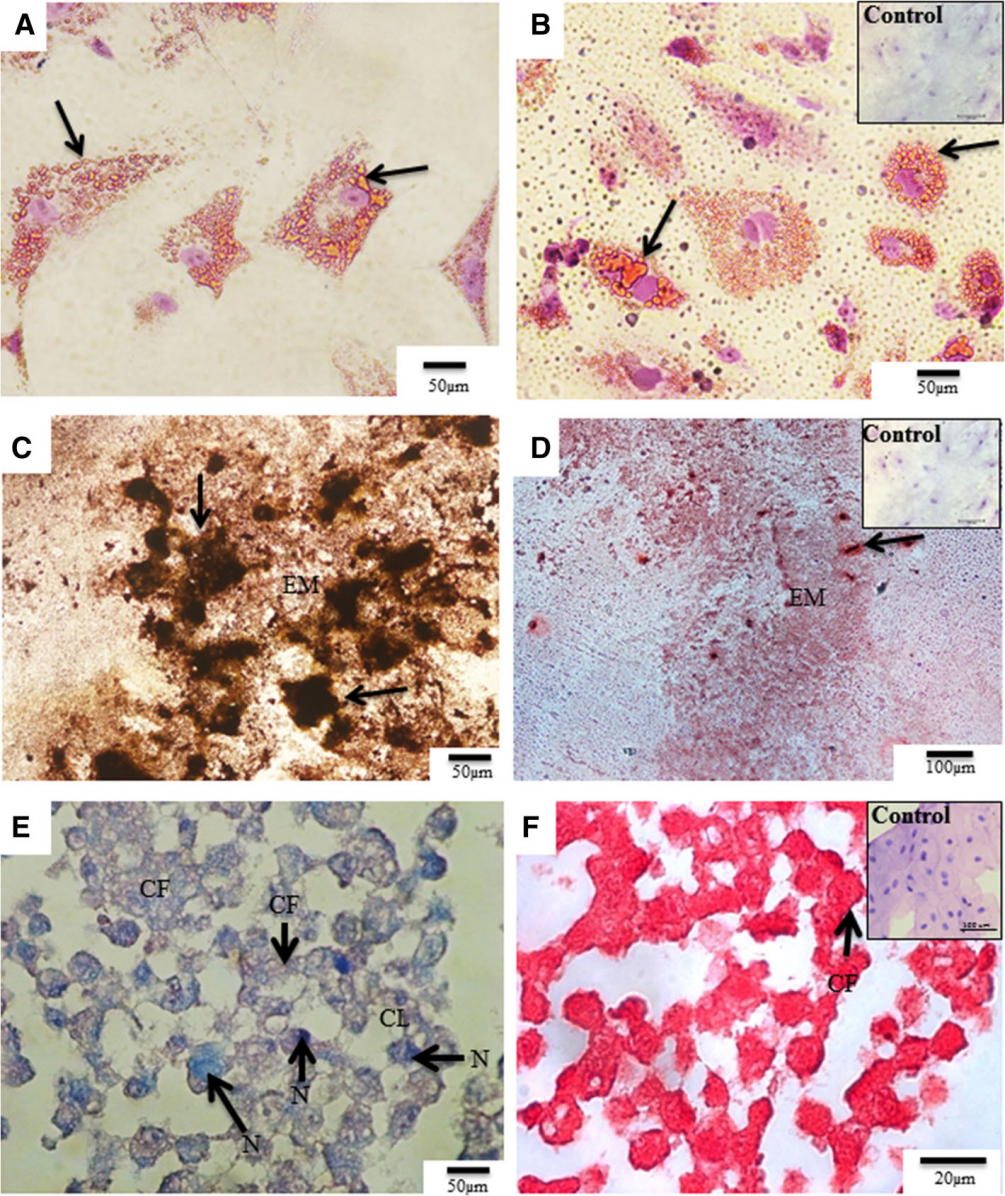
Differentiation assays (adipogenic, osteogenic and chondrogenic lineages) of cells isolated from the rabbit amniotic membrane. **a**, **b** Adipogenic differentiation, as shown by Oil Red staining. Lipid vesicles in the cytoplasm of cells (*arrows*). In addition, the nucleus had moved to the periphery of the cell. **c**, **d** Osteogenic differentiation as shown by Von Kossa and Alizarin Red staining, respectively. Note the presence of calcified regions (*arrows*) in the extracellular matrix (*EM*). **e**, **f** Chondrogenic differentiation, as shown by Masson’s Trichrome and Picrosirius staining, respectively. Cells showed a rounded shape with a globular nucleus (*N*) more strongly stained by Masson’s Trichrome which were surround by an extensive matrix rich in collagen fibres (*CF*), similar to the morphology of chondroblasts. In addition, there was formation of chondrocyte lacunae (CL)

After 21 days of induction, the amniotic membrane cells showed the ability to differentiate into osteogenic, adipogenic and chondrogenic lineages. During adipogenic differentiation, the fibroblast-like morphology of the cells was replaced by cells with a globular cytoplasm and a nucleus located at the periphery. Within the cytoplasm, clusters of lipid vesicles were observed after staining with Oil Red (Fig. 5a, b). During osteogenic differentiation, the formation of a calcified extracellular matrix was observed following staining by Von Kossa and Alizarin Red (Fig. 5c, d). Regions with greater calcification could be identified in the formed bone matrix. Differentiated cells of the chondrogenic lineage were stained by Trichrome Masson (Fig. 5e) and Picrosirius (Fig. 5f). We noted that the cells lost their previous fibroblast-like morphology and adopted a more rounded shape, with the appearance of regions similar to chondrocyte lacunae and the presence of collagen fibres (Fig. 5e, f).

### Tumourigenicity assay

**Fig. 6:**
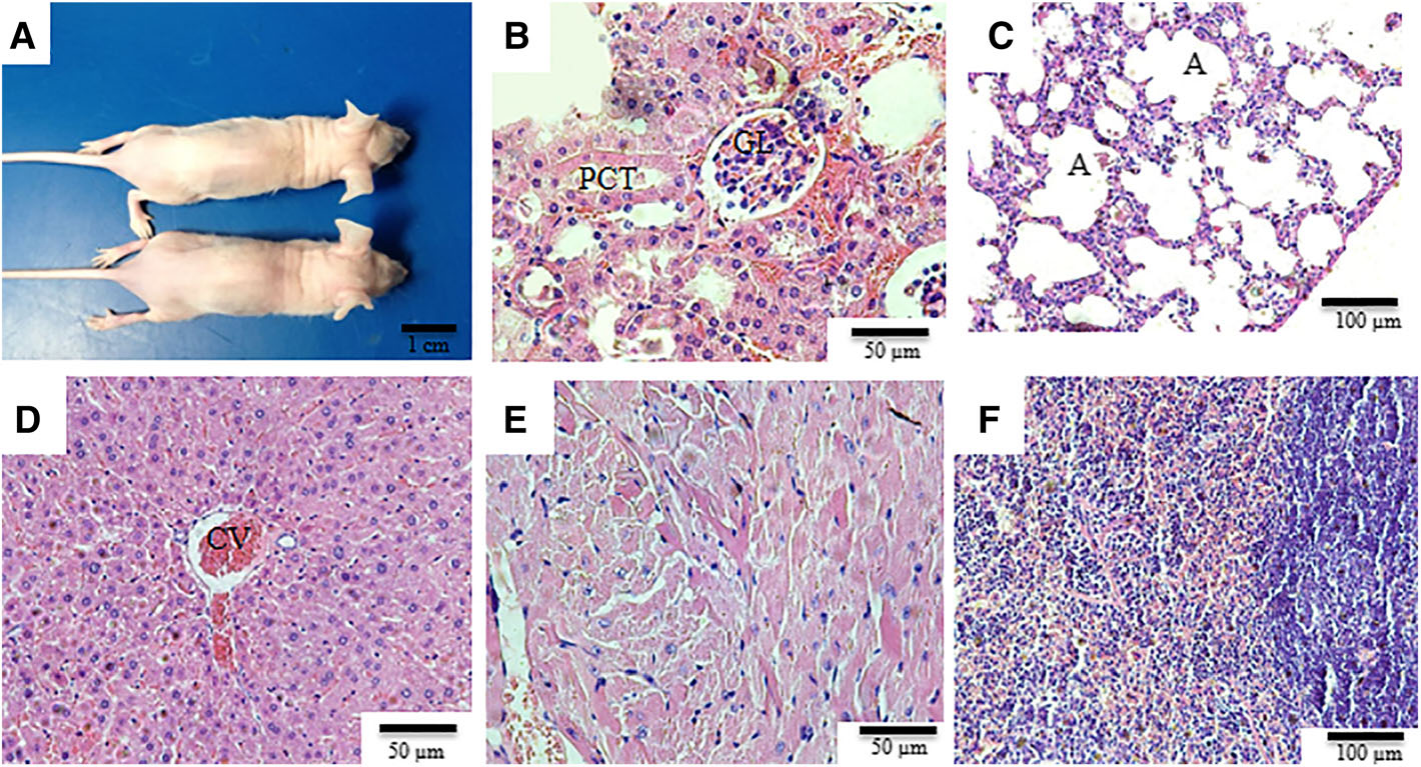
Tumorigenic analysis of rabbit amniotic membrane cells injections into nude mice. **a** Macroscopically, after 8 weeks of injection there was no tumour formation. Histopathologic analysis of the **b** kidney, **c** lung, **d** liver, **e** heart and **f** spleen. In all samples analysed, there was no evidence of tumour formation, and the organs were observed to have retained normal histological features. *GL* glomerulus, *PCT* proximal convoluted tubule, *A* alveolus, *CV* lobular central vein

After 8 weeks, the nude mice, which were evaluated clinically every week, did not show any signs of macroscopic or histopathological tumour formation. For further analysis, the animals were euthanized, and the mice receiving cell injections were analysed in detail (Fig. 6a). Histopathologically, the kidneys, lungs, liver, heart and spleen were analysed, showing normal structural characteristics with no changes that were related to metastatic tumour growth (Fig. 6b–f).

## Discussion

Recent studies have demonstrated a significant differentiation capacity for stem cells derived from the amniotic membrane, reaffirming the importance of studying the morphology, cell development, differentiation ability, applicability and in-vivo treatment potential of these cells. In the near future, it might be possible to extend the applicability of these cells to other diseases in addition to the cell therapy applications in which they have already been used [10, 41, 42]. Here, we investigated, for the first time, the capacity of the rabbit amniotic membrane to serve as a source of mesenchymal stem cells, with a focus on establishing a protocol for culturing these cells and characterizing these cells and their potential for differentiation using immunophenotyping.

First, we identified the mid-point of pregnancy (16 days) as the ideal pregnancy period for collecting the amniotic membrane, which is similar to what has been determined for rats [43]. Studies of the amniotic membrane [44] and umbilical cord blood [45] from early gestation and near term have suggested that cells obtained from early gestation display a higher proliferative activity and a higher level of pluripotency and mesenchymal stem cell marker expression compared with cells obtained late in gestation. It has been observed that cells isolated from the amniotic membrane during mid-gestation showed increased telomerase activity compared with cells from late gestation [43]. However, these studies also showed that, although there are differences in the phenotypes of the cells based on their gestational period, their immunomodulatory capacity remained unchanged. Based on the data available from the scientific literature and the ease with which the amniotic membrane can be separated from the other membranes at that time, the 16th day of gestation was used throughout this study.

Moreover, amniotic membrane cells were isolated using the explant method, which was the same as has been used for rats [27] and dogs [3], in contrast to other studies that used enzymatic digestion with trypsin or collagenase to obtain a more homogeneous cell culture [46, 47]. According to the method of isolation of cells from amniotic membrane, two cell types can be obtained: epithelial cells can be isolated by trypsin digestion, while fibroblast cells can be isolated by collagenase digestion [10]. However, our culture was produced by the mechanical method (i.e. explants) and produced a homogeneous culture containing only fibroblastic-like cells. After defining the ideal isolation method, we next optimized the culture medium, because there is limited information available in the literature, particularly with regard to rabbit cells. The media most commonly used were ALFA-MEM [24], DMEM-F12 [7, 48] and, most often, DMEM-HIGH [21, 49, 50]. We tested all three different media, which differed in their glucose concentrations, to investigate their influence on cell culture.

Glucose is the energy source for all cells; however, high concentrations of glucose can affect proliferation, differentiation and apoptosis, and can induce cell senescence. The culture media tested had glucose concentrations ranging from 5.6 to 25 mM [51]. Human bone marrow stem cells were cultured using media with high (25 mM), middle (11 mM) and low (5.6 mM) glucose concentrations [52] to assess cell proliferation, and the results showed that when cells were exposed in a short-term culture (4 days), no effect was observed on cell proliferation. In contrast, when exposed to long-term culture (4 weeks), cells showed a slight but significant reduction in proliferation in the high-glucose medium. In our study, we observed that both ALFA-MEM (5.6 mM) and DMEM-F12 (17.6 mM) produced less satisfactory growth compared with DMEM-HIGH (25 mM). The DMEM-HIGH condition produced improved growth and proliferation in vitro of rabbit amniotic membrane-derived stem cells, which differed from the results of Li et al. [52]. It is noteworthy that the first adherent cells with a fibroblast-like morphology were observed in the first 24 hours of culture, which has also been shown for rat amniotic cells [27].

After the cell culture was established, we performed a cryopreservation assay. Cells were frozen and later thawed for analysis, and similar fibroblast-like morphologies and undifferentiated, high proliferative capacities were observed, which is similar to what has been previously demonstrated for cells derived from cats [7]. Therefore, it is possible to create a cell bank for expansion and to perform other future experiments such as in-vitro and in-vivo tests.

In our cell viability studies, we observed using the MTT assay that the cells during passage 4 showed an initial increase in cell growth followed by a decrease before starting to grow again before a final decrease at the end of the experiment. As shown in Fig. 1e and f, when we used cells from passage 8 we observed a continuous pattern of growth that was followed by a decrease until the end of the experiment. These findings corroborate previous data observed from cell culture, showing that cells from passage 4 have an increased proliferation and expansion capacity overall compared with cells from advanced passages (passage 8); although the latter passage cells show continuous proliferation, it is delayed relative to the early passages. This finding is in contrast to other data from fibroblast cells obtained from the cat amniotic membrane, which showed a continuous growth throughout the entire experiment [7]. However, our results are more similar to data obtained using cell cultures [53]. In this previous study, the data showed an initial wave of proliferation that was followed by a drop in the proliferation rate, which ultimately rebounded at the end of the experiment.

Subsequently, we analysed the immunophenotypes of the cells during passages 4 and 8 by flow cytometry and observed significant differences in the two passages in the expression of various markers. We evaluated cytoskeletal and mesenchymal stem cell markers such as vimentin and cytokeratin 18, which are intermediate filaments, and β-tubulin, a microtubule membrane protein. Both vimentin and cytokeratin showed high levels of expression in the amniotic stem cells, demonstrating that our culture was heterogeneous in the cellular composition of mesenchymal and epithelial cells [47]. We assessed markers for mesenchymal stem cells such as CD73, CD105 and Stro-1, which also showed positive staining in our cultures. The mesenchymal stem cell marker CD105 had high expression during both passages 4 and 8 (40% and 47.9%, respectively). Similar results have been obtained with cells from humans [54]. However, Stro-1 showed a decreased expression during passage 8 (40.1%) compared with passage 4 (58%), which could be explained by the fact that one of the characteristics of this marker is its loss of its expression during subsequent passages [55]. However, the expression of CD73 during passage 4 was almost negligible (3.72%), while during passage 8 there was a considerable increase in its expression (35.7%) [5, 7, 21]. The rabbit amniotic cells were also positive for the haematopoietic precursor cell marker CD117 and were negative for the haematopoietic marker CD45, which is in agreement with previous studies [7, 27, 50].

PCNA-3, a marker of proliferating cells, was increased by approximately 9.7% in passage 4 compared with passage 8. This is in agreement with our culture and MTT analyses, which showed that the early passages were more proliferative compared with later passages, which is similar to what has been observed in the sheep amniotic membrane [44]. As demonstrated previously in other species [11, 26, 48], rabbit amniotic stem cells showed high levels of expression for pluripotency markers such as SSEA-4 and Sox-2. In contrast, Nanog and TRA-1 were highly expressed in the passage 4 cells but were expressed at insignificant levels in passage 8 cells (2.73% and 2.76%, respectively). The *Nanog* is responsible for maintaining the pluripotency and the self-renewal characteristics of embryonic stem cells, but the factors that control Nanog expression remain unknown [56]. TRA-1 is expressed on the surface of embryonic cells and is downregulated during differentiation. TRA-1 is also similarly expressed in embryonic carcinoma cells [57]. In addition, in both passages 4 and 8 the rabbit amniotic stem cells expressed the genes *c-myc* and *Lin28a* in the RT-qPCR analysis, which are involved with pluripotency. These proteins were studied previously in amniotic stem cells obtained from the human and bovine amnion [58, 59]. Therefore, we can conclude from these results that the rabbit amniotic membrane cells exhibit greater marked pluripotency characteristics in passage 4 compared with passage 8. The ability of cells to differentiate into specific lineages is one of the criteria for their classification as stem cells. In our study, we induced the cells through exposure to specific media to differentiate into the adipogenic, osteogenic and chondrogenic lineages. Rabbit amniotic stem cells were able to differentiate into all three cell lineages, which was expected based on similar results in cells from the human [10, 21, 30, 60], horse [49], cat [7, 26] and dog [5] amniotic membranes. In this study, the time necessary for differentiation was 21 days, which was the same as in the references cited previously. However, this differs from stem cells derived from adult tissue, where the differentiation time is shorter (14 days) [61, 62]. The time needed for cellular differentiation is directly related to the source of the cells being induced. In general, adult tissue stem cells are committed to differentiate into one specific cell type, and this is why a shorter time is required for differentiation. Many studies have emerged to attempt to explain the effects of high concentrations of glucose on cell differentiation. In this context, high glucose levels result in the induction of osteogenic differentiation [63]. In that study, the authors tested various concentrations ranging from 5.5 to 35 mM, and their results demonstrated that high concentrations of glucose suppressed the bone morphogenetic protein-2 (BMP-2) signalling pathway, which plays an important role in the regulation of osteogenic cell differentiation. The authors concluded that high concentrations of glucose decrease the ability of cells to differentiate along the osteogenic lineage. However, in a similar study, two different concentrations (17.6 and 25 mM) were tested, and the results showed that the cells were able to differentiate along the osteogenic lineage only when higher glucose concentrations were used [52]. This latter finding is similar to our results, because we used a medium containing a high concentration of glucose (DMEM-HIGH, 25 mM) for the induction of osteogenic differentiation from rabbit amniotic membrane cells. For adipogenic differentiation, it has been demonstrated that high glucose concentrations increase both differentiation along the adipogenic lineage and lipid accumulation [64]. This is similar to our results where the use of DMEM-HIGH (25 mM) produced satisfactory adipogenic differentiation with the detection of fat vesicles inside the cytoplasm. Expanded cells cultured in high glucose concentrations prior to differentiation have been shown to have a decreased ability for chondrogenic differentiation [65, 66]. Conversely, Han et al. [67] showed that a high glucose concentration (30 mM) had a dose-dependent stimulatory effect on chondrogenesis. In this study, the cells used to assess chondrogenic differentiation were previously grown in DMEM-HIGH, which had the highest glucose concentration of the media tested and was similar to the medium described by Han et al. [67]. Using this medium, we were able to obtain a satisfactory differentiation of amniotic cells into chondrocytes.

In this field, studies have demonstrated that lysophosphatidic acid (LPA), an extracellular lipid molecule, is involved with the maintenance of cell survival and proliferation in vitro. Originally produced in reproductive tissues, LPA controls the ovarian cycle and pregnancy, acting in the uterus, ovary, placenta and foetal membranes [68]. In addition, LPA expression is higher in amniotic membrane than in placental tissues [69]. Kim et al. [70] reported that when stimulated with LPA the amniotic membrane cells increased cell proliferation, similar to what is known for neuronal and blood stem cells [71, 72].

Following the isolation, characterization and differentiation of rabbit amniotic cells, which are prerequisites to define a stem cell, we conducted a tumourigenic potential assay to evaluate the safety of these cells for use in regenerative medicine applications. The rabbit amniotic cells from both passages 4 and 8, when injected into nude mice, did not show metastatic potential. This result confirms previous descriptions regarding the safety of amniotic stem cells [7, 53].

## Conclusion

We were able to obtain a multipotent mesenchymal stem cell lineage from the rabbit amniotic membrane. These cells expressed specific markers for mesenchymal stem cells and pluripotency, did not express markers of haematopoietic cells and were able to differentiate into different mesenchymal cell lineages. In addition, the analysis of tumourigenic potential showed that rabbit amniotic stem cells are safe for use in future therapies, as has been previously demonstrated for other species. These cells also exhibit a high level of expandability and the capacity for proliferation in vitro, which are essential characteristics for storage in cell banks.
